# Vitreous Hemorrhage Case Report

**DOI:** 10.21980/J88D3B

**Published:** 2022-07-15

**Authors:** Mary Rometti, Laryssa Patti, Christopher Bryczkowski

**Affiliations:** * Rutgers Robert Wood Johnson Medical School, Department of Emergency Medicine, New Brunswick, NJ

## Abstract

Ocular issues are a common reason to present to the emergency department (ED). This case discusses a patient who presented to the ED with unilateral atraumatic partial vision loss. The patient underwent a point of care ultrasound that was concerning for a vitreous hemorrhage. Although vitreous hemorrhages require urgent, rather than emergent evaluation, it is important to differentiate this diagnosis from vitreous and retinal detachment.

**Topics:**

Vitreous hemorrhage, eye complaint, point of care ultrasound, POCUS.

**Figure f1-jetem-7-3-v20:**
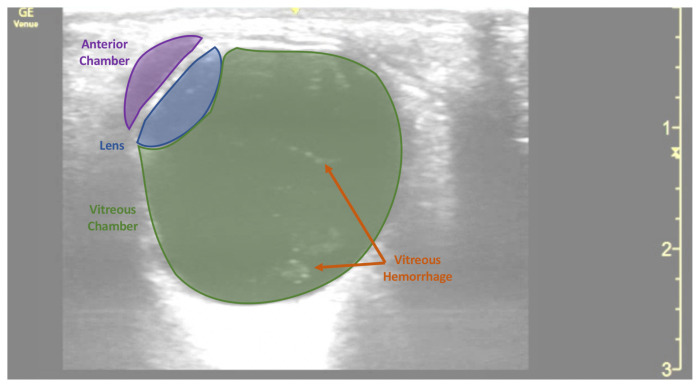
Overgained Ultrasound Video Link: https://youtu.be/bjpWj2b1QjY [Fig f1-jetem-7-3-v20]Ultrasound Video Link: https://youtu.be/LYyN-I0irDs

## Brief introduction

Approximately 2–3% of all emergency department (ED) visits are related to ocular complaints.^1^–^4^ Differentiating between vitreous hemorrhage, vitreous detachment, and retinal detachment is important because retinal detachments are ophthalmological emergencies that may necessitate emergent ophthalmology evaluation.^1^,^3^ Because many EDs do not have ophthalmologists who are readily available for emergent consultation, some conditions may necessitate transfer to another institution for emergent evaluation by an ophthalmologist.^2^ Ultrasound offers a unique way to efficiently examine the eye from the patient’s bedside to help evaluate urgent versus emergent ophthalmologic diagnoses and begin narrowing down the differential.^1^–^3^,^5^

## Presenting concerns and clinical findings

A 63-year-old male with a past medical history of hypertension presented to the ED with one week of painless, partial vision loss in his left eye, which he described as a black spot in the center of his vision. He denied headache, dizziness, eye discharge, or trauma. He wears glasses for reading and does not wear contact lenses.

On physical exam, there was a loss of red reflex in his left eye. His pupils were equal and reactive to light with extraocular movements intact. Visual acuities were 20/40 for the right eye, 20/50 for the left eye, and 20/30 for both eyes.

## Significant findings

Point of care ultrasound (POCUS) revealed hyperechoic material in the vitreous consistent with a vitreous hemorrhage. On the ultrasound images, there is visible hyperechoic debris seen floating in the vitreous as the patient moves his eye. Since the vitreous is typically anechoic (black) in color on ultrasound, turning up the gain on the ultrasound machine makes these findings easier to see and often highlights abnormalities, such as this hemorrhage (see annotated still).

## Patient course

In the ED, ophthalmology was consulted. Arrangement was made for the patient to follow-up in clinic that same day for further ophthalmologic care. The patient was seen in ophthalmology clinic that day, and the diagnosis of vitreous hemorrhage was confirmed.

## Discussion

Typically, patients with vitreous hemorrhages will report unilateral painless visual changes.^6^ If the presentation is associated with trauma, orbital pain and pain with eye movements may also be present.^6^ Physical exam may show a decreased red reflex.^6^

Vitreous hemorrhages occur when blood extravasates into the space located between the lens and the retina, which can occur from normal blood vessels impacted by mechanical forces or from pathologic blood vessels as with diabetic retinopathy.^6^,^7^ A patient’s presentation often depends on the amount of hemorrhage in the vitreous.^6^ Visual changes have been described with as little as 12.5 microliters of blood.^6^ Generally, an ultrasound with a vitreous hemorrhage will show hyperechoic debris in the vitreous which may move with ocular movements.^2^–^5^,^8^ POCUS has a sensitivity of 81.9% and a specificity of 82.3% for diagnosing vitreous hemorrhage.^1^,^6^ Since these findings may be subtle and difficult to notice in otherwise anechoic vitreous, increasing the gain setting on the ultrasound may help in visualizing these findings.^2^

In the emergency department, an ocular ultrasound can be performed for many indications: ocular trauma, visual changes or loss, ocular pain, or concern for increased intracranial pressure.^2^ If there is concern for a globe rupture, an ocular ultrasound ideally should not be performed because it is a relative contraindication.^2^

Generally, an ocular ultrasound is performed using a high frequency transducer secondary to its ability to generate a higher resolution image of superficial structures.^3^ One of the most common techniques to performing an ocular ultrasound involves placing a transparent adhesive film dressing over the patient’s closed eyelid with ultrasound gel on top of the dressing.^2^,^4^ A high-frequency linear probe can then be used to scan the eye in transverse and sagittal views to examine the entire orbit.^2^,^3^

Management for vitreous hemorrhages focuses mainly on ensuring the patient has appropriate follow-up with ophthalmology, ideally within 24 hours.^6^ Discovering the underlying cause and treating it should resolve the vitreous hemorrhage.^6^ The clearance of the blood in the vitreous is estimated at about 1% per day.^7^ Patients should also receive instructions on symptomatic treatment.^6^

If not treated appropriately, progression of vitreous hemorrhages may result in retinal detachment, glaucoma, or permanent vision loss.^6^ More invasive treatment, such as a vitrectomy, is generally only recommended for vitreous hemorrhages that do not resolve.^7^ Anticoagulation is not an associated risk factor for vitreous hemorrhage development if there is no coagulopathy.^6^ However if a patient is taking warfarin, it is reasonable to check the international normalized ratio (INR).^6^

This case demonstrates the importance of utilizing POCUS in being able to recognize and effectively treat a vitreous hemorrhage in a patient presenting to the Emergency Department with visual decline. The use of ultrasound allowed the physician to rapidly differentiate among different eye emergencies and provided timely, appropriate follow up treatment. A limitation of this case is that the ultimate diagnosis and additional follow-up arranged by ophthalmology is not known because the patient was only followed in the ED. While not all ocular complaints are critical, recognizing the emergent from the less time-sensitive diagnoses is vital in the Emergency Department.

## Supplementary Information








