# Supplementary figures and images for: Vitreous Hemorrhage Case Report
Source: J Educ Teach Emerg Med. 2022 Jul 15;7(3):V20–2. doi: 10.21980/J88D3B (PMC10332704; doi:10.21980/J88D3B)

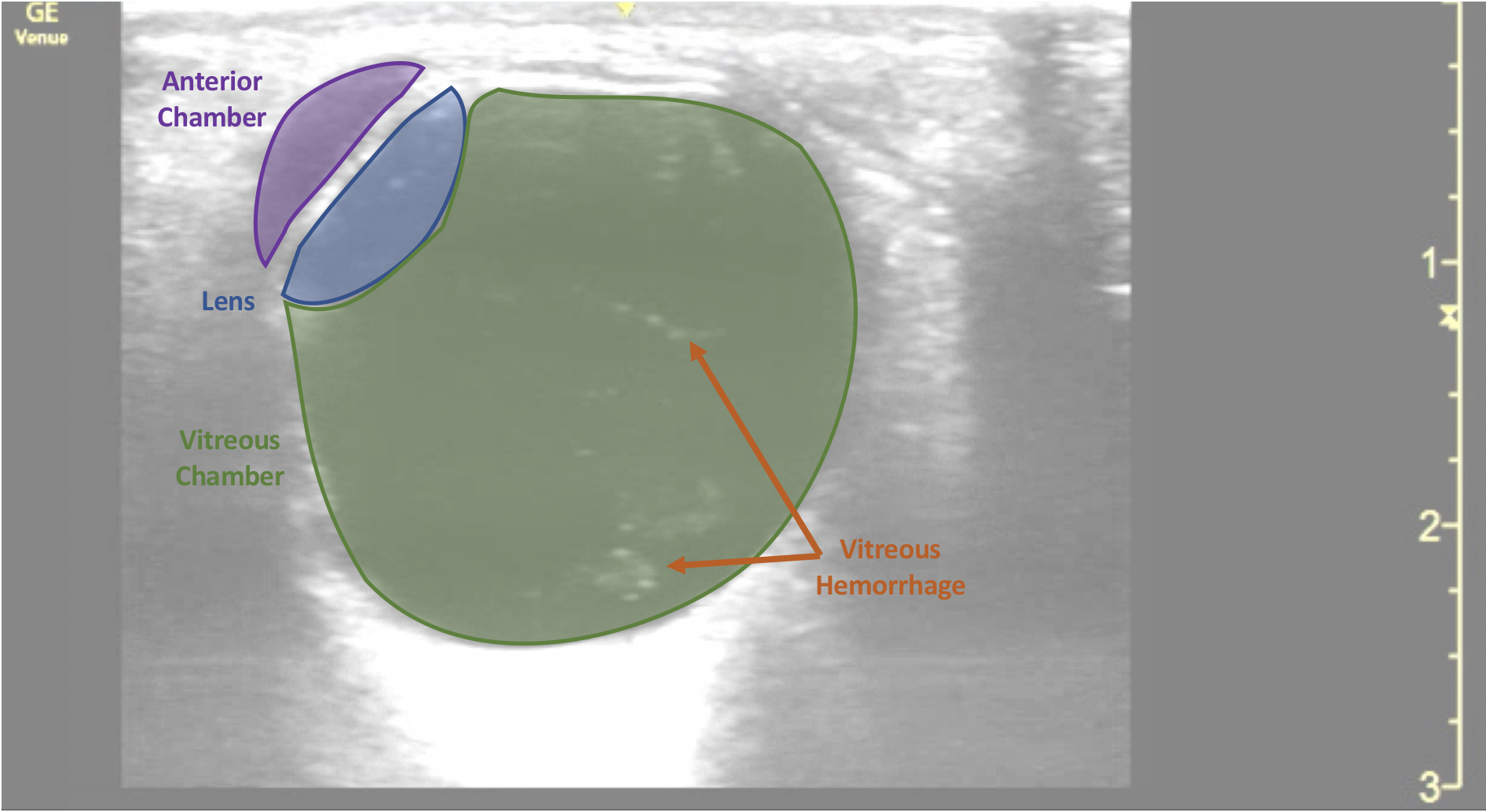

Supplement: Supplementary file 3 [file jetem-7-3-v20-supp3.jpeg]

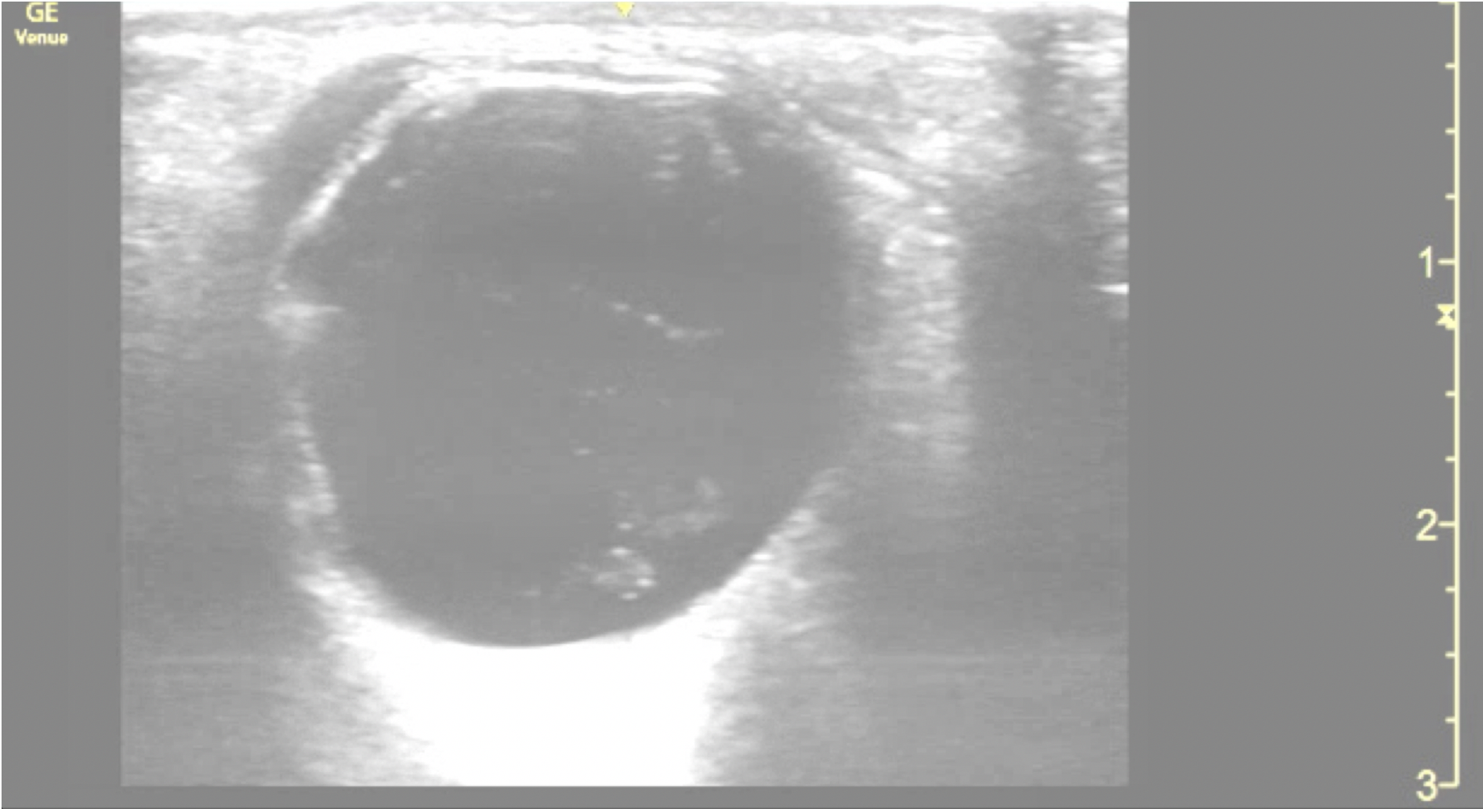

Supplement: Supplementary file 4 [file jetem-7-3-v20-supp4.jpeg]
